# Digital Image Analysis Applied to Tumor Cell Proliferation, Aggressiveness, and Migration-Related Protein Synthesis in Neuroblastoma 3D Models

**DOI:** 10.3390/ijms21228676

**Published:** 2020-11-17

**Authors:** Ezequiel Monferrer, Sabina Sanegre, Susana Martín-Vañó, Andrea García-Lizarribar, Rebeca Burgos-Panadero, Amparo López-Carrasco, Samuel Navarro, Josep Samitier, Rosa Noguera

**Affiliations:** 1 Department of Pathology, Medical School, University of Valencia-INCLIVA Biomedical Health Research Institute, 46010 Valencia, Spain; ezequiel.mo.ga@gmail.com (E.M.); Sabina.sanegre@gmail.com (S.S.); Susana.Martin@uv.es (S.M.-V.); reburpa@alumni.uv.es (R.B.-P.); amparolopezcarrasco@gmail.com (A.L.-C.); samuel.navarro@uv.es (S.N.); 2 Low Prevalence Tumors, Centro de Investigación Biomédica en Red de Cáncer (CIBERONC), Instituto de Salud Carlos III, 28029 Madrid, Spain; 3 Institute for Bioengineering of Catalonia, Barcelona Institute of Science and Technology (IBEC-BIST), 08028 Barcelona, Spain; agarcial@ibecbarcelona.eu (A.G.-L.); jsamitier@ibecbarcelona.eu (J.S.); 4 Department of Electronics and Biomedical Engineering, University of Barcelona, 08028 Barcelona, Spain; 5 Networking Biomedical Research Center in Bioengineering, Biomaterials and Nanomedicine (CIBER-BBN), 28029 Madrid, Spain

**Keywords:** 3D cancer modeling, Ki67, vitronectin, DOCK8, KANK1, preclinical therapeutic studies

## Abstract

Patient-derived cancer 3D models are a promising tool that will revolutionize personalized cancer therapy but that require previous knowledge of optimal cell growth conditions and the most advantageous parameters to evaluate biomimetic relevance and monitor therapy efficacy. This study aims to establish general guidelines on 3D model characterization phenomena, focusing on neuroblastoma. We generated gelatin-based scaffolds with different stiffness and performed SK-N-BE(2) and SH-SY5Y aggressive neuroblastoma cell cultures, also performing co-cultures with mouse stromal Schwann cell line (SW10). Model characterization by digital image analysis at different time points revealed that cell proliferation, vitronectin production, and migration-related gene expression depend on growing conditions and are specific to the tumor cell line. Morphometric data show that 3D in vitro models can help generate optimal patient-derived cancer models, by creating, identifying, and choosing patterns of clinically relevant artificial microenvironments to predict patient tumor cell behavior and therapeutic responses.

## 1. Introduction

Despite advances in treatment, cancer remains one of the leading causes of death worldwide due to its complexity. Developing physiologically accurate cancer models is therefore essential to uncover the keys to cancer progression and test new therapeutic approaches. As explants of small tumor samples, traditional bi-dimensional (2D) cell cultures have been among the most widely used tools as in vitro models for biomedical research, due to their simplicity and low cost. However, they are incapable of reflecting the effect of the tumor microenvironment or the multiple different cellular interactions that take place in this three-dimensional (3D) environment [1]. The extracellular matrix (ECM), a vital part of the tumor microenvironment, is a 3D network present in all malignant tissues involved in different functions related to protection, nutrition, cell innervation, immune response, angiogenesis, fibrosis, and tissue regeneration [2,3,4,5]. It also participates in transmitting mechanical forces that support the tissue tensegrity system, activating cellular genetic and epigenetic mechanisms. ECM alterations can result in multiple dysfunctions [6], and although this network is not considered in standard cancer management practice today, there is growing evidence pointing to its key role in tumor progression and therapy resistance [7]. 

3D cell culture has attracted great attention in in vitro cancer modeling due to its ability to recapitulate the structural and functional aspect of the corresponding organs [1]. These models have already been used to study the impact of the ECM on different cancer cells, showing that ECM stiffness may enhance cell motility by modifying their morphological properties to an aggressive phenotype [8,9]. A variety of different tools and methods can be used to generate these 3D models depending on the desired characteristics [10]. Although 3D spheroids and organoids have been broadly used as cancer models for solid tumors, there is no scope for fine-tuning to modulate ECM composition and architecture. One of the most sophisticated methods to overcome the complexity and low reproducibility of 3D spheroids and organoids is 3D bioprinting technology, which allows direct and indirect cell incorporation into the bioink, accurate cell spatial distribution, and defined mesh structure patterning with the desired architectural features [11,12]. 

For many years, our research has focused on the role of the ECM in neuroblastoma (NB) [13,14,15,16] the most frequent extracranial solid tumor in childhood, typified by its highly variable prognosis [17]. A stiff ECM has been associated with NB aggressiveness, since malignant neuroblastic cells are highly sensitive to the biomechanical properties of their microenvironment [13,14,15,16,18]. Performing digital image analysis, we have successfully defined morphological patterns associated with ECM stiffness in NB, considering different components such as blood and lymph vessels, collagen fibers, glycosaminoglycans (GAGs), and glycoproteins such as fibronectin and vitronectin (VN) [13,14,15,16,19,20]. In recent years, we have also worked with 3D cell culture in bioprinted models, showing that the biomechanical forces imposed by the scaffolds have a great impact on malignant neuroblastic cell behavior [21,22]. 

The goal of translational research in oncology is to develop patient-derived cancer models to improve personalized therapy. This requires prior work with representative preliminary in vitro 3D models to establish and document the conditions modulating of the 3D cell culture behavior, as well as to assess drug response in preclinical therapeutic studies conducted on the most reliable models. In the present study, we generated 3D bioprinted gelatin-based models to perform 3D cell cultures. We selected two neuroblastoma cell lines with different genetic backgrounds and degrees of malignancy, with the previously used *MYCN*-amplified SK-N-BE(2) cells and *ALK*-mutated SH-SY5Y cells both representing high-risk neuroblastoma [23,24], and evaluated their behavior under different stiffness conditions. To develop a more physiologically relevant environment, we also co-cultured the NB lines with stromal Schwann cells (mouse Schwann cell line (SW10)). Including Schwann cells in the models allows the tumor cells to grow in a more biomimetic environment, since these cells are found at low proportions even in undifferentiated or poorly differentiated neuroblastoma with poor stroma, and are pivotal players in stroma synthesis, which helps determine aggressiveness in this pediatric cancer [25]. 

When choosing a model to carry out preclinical therapeutic studies, it is necessary to determine the optimal characteristics required from this model, taking into account the desired therapeutic approach and most useful related parameters to analyze. In this study, as a generic aggressiveness factor, we analyzed Ki67, a widely used marker for cell proliferation assessment in cancer studies [26,27]. Our previous studies highlighted territorial VN (tVN) as a relevant glycoprotein related to NB aggressiveness and stiffness [19,20]. VN also interacts with different ECM molecules and has an important role in metastasis through its RGD integrin binding domain, which allows tumor cells to migrate [28,29,30]. This makes VN a potential therapeutic target, assuming that inhibiting its interaction with tumor cell integrins and/or its ECM remodeling action could reduce tumor cell migration, which prompted us to evaluate its presence in 3D NB models. We also studied the expression dynamics of two additional genes, DOCK8 and KANK1, having detected a common atypical segmental chromosomal aberration affecting these genes located in chromosome 9p in some high-risk NB patients, as well as in orthotopic mice models and 3D NB bioprinted models [22]. DOCK8 is involved in cytoskeleton remodeling, inducing migration in macrophages, Schwann precursors cells, T cells, and dendritic cells [31,32,33,34]. KANK1 participates in actin and microtubule regulation; however, its role in inhibiting or promoting migration depends on the system studied [35,36,37,38]. It has also been reported to play a role as a tumor suppressor gene [39,40].

In this study, we used morphometric digital analysis to identify cell characteristics and evaluate cell behavior on 3D scaffold-based NB models. Following on from our previous genetic studies [22], we have chosen replicates of the 3D cultured samples to characterize the expression pattern of the above-mentioned markers and pinpoint correlations with their genomic landscape. The specific parameters analyzed herein provide proof of concept of the wide spectrum of possibilities that these techniques offer to obtain clinically relevant in vitro models for preclinical therapeutic assessment.

## 2. Results

**Figure 1:**
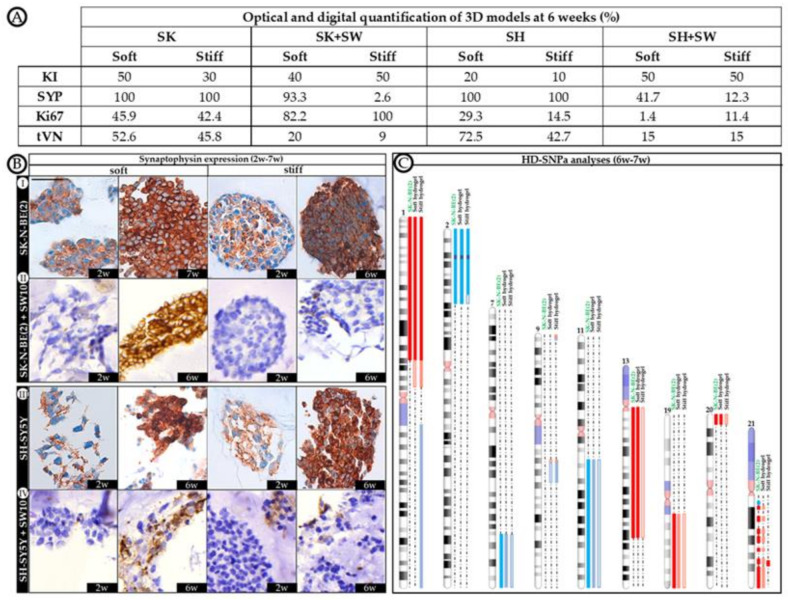
Morphological and genetic characterization of 3D models. (**A**) Optical and digital quantification of 3D models at 6 weeks. Quantifications were performed by digital image analysis, except for the karyorrhexis index (KI), which was obtained by optic microscopic assessment. Proportion of neuroblasts (data of neuroblasts in co-culture were obtained removing the proportion of non-expressing synaptophysin cells, as we considered them SW10 cells) expressing synaptophysin (SYP), Ki67, tVN (territorial vitronectin strong staining expression) are detailed. (**B**) Immunostaining of synaptophysin in 3D models at short (2 weeks) and long (6–7 weeks) cell culture times. Scale bar 25 µm at top left of the first image. Same scale bar is valid for all images. (**C**) Genomic analyses. Schematic representation of the chromosomal aberrations differentially detected by HD-SNPa in soft and stiff hydrogels cultured with SK-N-BE(2) cell line over 6 weeks, and compared with SK-N-BE(2) cell line grown in 2D. For each altered chromosome, gains of genomic material are represented in blue, deletions in red, and *MYCN* amplification in purple. As the proportion of cells affected by the chromosomal aberrations decreases, their color becomes lighter. No genomic differences were observed between hydrogels grown with SK-N-BE(2) cell line alone and co-cultured with SW10 cell line in any of the studied conditions. Genomics of SH-SY5Y cells remained stable in every studied 3D condition, identical to that of 2D cultures [22].

To assess whether 3D culture generates cell behavioral changes, we first characterized the three cell lines individually in 2D cultures. SK-N-BE(2) and SH-SY5Y cell lines presented Ki67 positivity (80 and 50%, respectively), and KANK1 was detected in all cells. However, DOCK8 staining was found to be negative (<1%) in these cell lines, and little VN was present in most cells (70%), although with different expression patterns; cell membrane and staining in neuritic prolongations in SK-N-BE(2) and perinuclear cytoplasmic staining in SH-SY5Y. SW10 cells presented low Ki67 positivity (<1%), but all cells were DOCK8 and KANK1 positive. VN was negative in SW10 cultures (<1%). Furthermore, to shed more light on cell behavior, synaptophysin (SYP) expression was used as a differential marker between SW10 and neuroblastic cells at 6 weeks in 3D cultures, since both SK-N-BE(2) and SH-SY5Y cell lines were 100% positive and SW10 negative (Figure 1B).

### 2.1. Differential Effect of 3D Hydrogel Stiffness on Cell Proliferation in SK-N-BE(2) and SH-SY5Y NB Cell Lines

**Figure 2:**
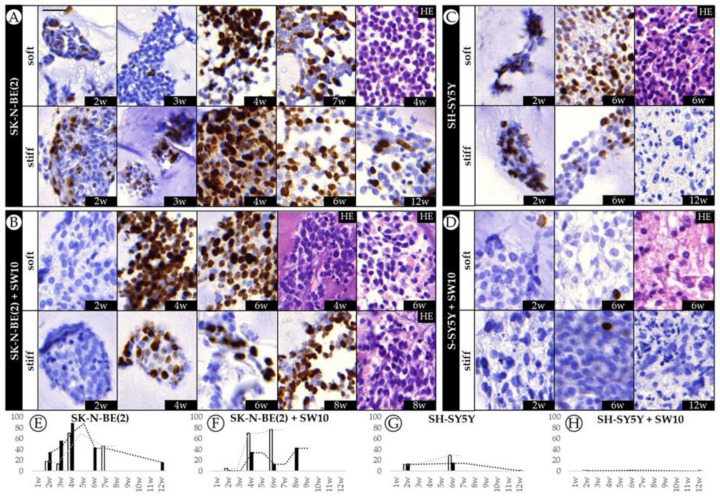
Dynamics of SK-N-BE(2) cell and SH-SY5Y cell proliferative indices over time. (**A**–**D**) Representative images of Ki67 expression at the time points studied (w: weeks) and hematoxylin eosin (HE) for each cell culture/co-culture in soft and stiff scaffoldings. The images on the left correspond to the SK-N-BE(2) cell line cultivated (**A**) alone and (**B**) with mouse Schwann cell line (SW10); the images on the right represent the SH-SY5Y cell line cultivated (**C**) alone and (**D**) with SW10 cells. Scale bar 25 µm at top left of the first image. Same scale bar is valid for all images. (**E**–**H**) Bar chart quantification of Ki67 staining (% of positive cells) for (**E**) SK-N-BE(2) cells and (**F**) SK-N-BE(2) cells plus SW10 cells in soft and stiff scaffolds, and for (**G**) SH-SY5Y cells and (**H**) S-SY5Y cells plus SW10 cells in soft and stiff scaffolds. White and black bars: soft and stiff scaffolds, respectively. Dashed lines indicate moving average per stiffness condition. X axis: time in weeks (w) and Y axis: % of Ki67 positive cells.

With the aim of characterizing the long-term effect of biomechanical properties on tumor aggressiveness, we cultured two different cell lines over long time spans in soft and stiff gelatin-based hydrogels to evaluate proliferation dynamics over time. We have previously shown that scaffolding stiffness increased SK-N-BE(2) cell proliferation during the 2nd to 4th week of culture [21]. Immunohistochemistry (IHC) analysis showed that proliferation dynamics differ completely from one cell line to another. SK-N-BE(2) cells appeared to be far more proliferative than SH-SY5Y cells in any condition studied, with a Ki67 proliferative index of 88.1% in stiff conditions at 4 weeks (Figure 2A). In particular, we could observe heightened proliferation of SK-N-BE(2) cells from the 2nd to 4th week (with proliferation indices of 17.9 to 70.1% and 34 to 88.1% for soft and stiff hydrogels, respectively), as previously described. Furthermore, as reported, this increase in proliferation was dependent on the stiffness of the substrate, with the neuroblasts on stiff hydrogels showing greater proliferation. Interestingly, we have now noticed that the SK-N-BE(2) cell proliferative index decreased from the 4th–5th week of culture, although this cell line remained proliferative even at the 12th week (15.7%). In comparison, SH-SY5Y cells displayed lower proliferative indices than SK-N-BE(2) cells, as already observed in 2D cultures (50 and 80% respectively), reaching up to 29.3% of proliferative cells in soft conditions at 6 weeks and with little proliferation observed after 12 weeks of culture (0.2%) (Figure 2C). SH-SY5Y cells in soft hydrogels achieved higher proliferative indices than in the stiffer ones, as opposed to SK-N-BE(2) cells. 

### 2.2. The Contribution of Co-Cultured Stromal Schwann Cells to SK-N-BE(2) Proliferation Is Dependent on Substrate Stiffness

To recreate a more biomimetic tumor microenvironment, we co-cultured SK-N-BE(2) and SH-SY5Y NB cells with 10% Schwann cells and studied the contribution of the latter to NB cell line progression. Adding Schwann cells to SK-N-BE(2) cell cultures reduced proliferative indices in stiff hydrogels, while under soft hydrogel growth conditions the trend remained similar to that observed without co-culture (Figure 2B). However, the presence of Schwann cells in SH-SY5Y cell cultures hindered model proliferation, with 1.4% of Ki67 positive cells being the highest value observed across the time points studied (Figure 2D). Based on the SYP-positive population, we were able to determine the proportion of neuroblasts to total cells in co-cultured models from 6 weeks onwards, when genetic changes become more evident (Figure 1C). Furthermore, we determined the effect of SW10 cells on neuroblast proliferation according to the proportion of Ki67 positive neuroblasts (Figure 1A). Characterizing the neuroblast proportion in co-cultures at 6 weeks, we found that SK-N-BE(2) cells overtook the SW10 population proportionally in soft conditions (69.4% of neuroblasts). However, in stiff conditions, the proportion of neuroblasts remained very low (2.6%) and only overtook the Schwann cell population from 8 weeks onward (86.9% of neuroblasts). 

Analyzing the specific effect of SW10 incorporation on neuroblast proliferation, we observed that SW10 increased the SK-N-BE(2) proliferation capacity (Figure 1A). Moreover, substrate stiffness determined the degree of proliferation in SK-N-BE(2) co-cultures, since only co-cultured neuroblasts in stiff conditions achieved full proliferation capacity (42.4% expected vs. 100% observed). SW10 cells reduced SH-SY5Y cell proliferation when co-cultured, especially in soft conditions (29.3% expected vs. 1.4% observed) and also reduced the proportion of neuroblasts (Figure 1A).

### 2.3. Correlation between Mitosis–Karyorrhexis Index and Cell Number Differs with Growth Conditions

**Figure 3:**
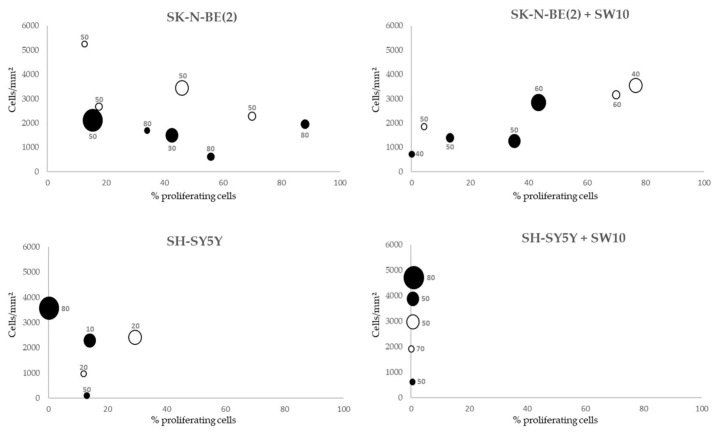
Correlation between proliferative index, cell number, and karyorrhexis index of each cell composition model and time of culture. White circles: soft models. Black circles: stiff models. Karyorrhexis index is shown for each culture condition. X axis: %Ki67 positive cells. Y axis: total number of cells/hydrogel area (mm^2^). Circle size is proportional to culture time (2 to 12 weeks).

The cell proliferative index (Ki67 positivity) and cell number (number of cells, standardized by hydrogel area present at the slides) were established for comparison between conditions. In order to assess cell death, we determined the karyorrhexis index (the percentage of necrotic and apoptotic cells). Cell detachment was confirmed by the high number of cells found growing on the well surface when culture times were stopped in all conditions. In SK-N-BE(2) 3D hydrogels, as the proliferative index increased, the number of cells tended to stabilize. However, with similar proliferative indices, the number of cells was higher in soft than in stiff condition. In SK-N-BE(2) cell plus SW10 cell hydrogels, in general, there was a positive correlation between proliferation and cell number (the greater the proliferation, the higher the number of cells) (Figure 3). Interestingly, there was a correlation between the time and cell number, but not with proliferation, in both SH-SY5Y cells alone and SH-SY5Y cells plus SW10 cells: the longer the hydrogel culture time, the more cells were present. As opposed to SK-N-BE(2) models, stiffness increased the number of cells in SH-SY5Y cells alone and SH-SY5Y cells plus SW10 cells models. Finally, we found that high proliferative indices were observed in SK-N-BE(2) both alone and co-cultured with SW10 did not yield an increase in the number of cells when compared with SH-SY5Y cultures. 

According to the karyorrhexis index, SK-N-BE(2) cells presented a higher proportion of cell death (55±6%) than SH-SY5Y cells (36±13%) in their respective models, with the former reaching their maximum index in stiff conditions at the short cell culture time (80%). Furthermore, although the karyorrhexis index showed slight variations between both SK-N-BE(2) cell and SK-N-BE(2) cells and SW10 cell models in the different conditions, this index increased in SH-SY5Y cells plus SW10 cells models compared with SH-SY5Y cells models (Figure 3). 

### 2.4. Differential 3D Culture Stimulation of tVN Expression Is Dependent on Cell Growth Conditions

Given that tVN is associated with an aggressive NB phenotype and proposed as a drug target, we aimed to characterize its dynamics over time. IHC results showed that tVN (high intensity staining) was greatly expressed in the cytoplasm and close pericellular space secreted by neuroblastic malignant cells in all 3D hydrogels regardless of their scaffold characteristics or cell composition, as opposed to 2D culture, where only neuroblasts expressed low cytoplasmic staining for VN. Interterritorial VN (low intensity staining) was considered as the ECM. 

**Figure 4:**
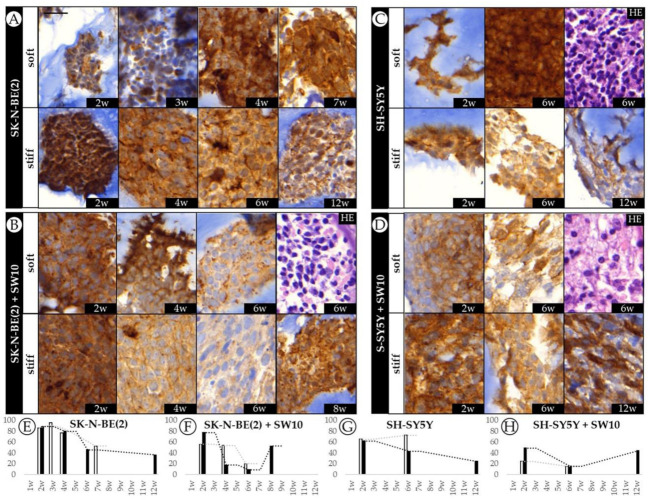
Dynamics of VN expression in SK-N-BE(2) and SH-SY5Y cell lines over time. (**A**–**D**) Representative images of VN expression at the time points studied (w: weeks) and hematoxylin eosin (HE) for each cell culture/co-culture in soft and stiff scaffoldings. The images on the left correspond to the SK-N-BE(2) cell line cultivated (**A**) alone and (**B**) with SW10; the images on the right represent the SH-SY5Y cell line cultivated (**C**) alone and (**D**) with SW10 cells. Scale bar 25 µm at top left of the first image. Same scale bar is valid for all images. (**E**–**H**) Bar chart quantification of VN staining (% of stained area) for SK-N-BE(2) cells (**E**) alone and (**F**) SK-N-BE(2) cells plus SW10 cells in soft and stiff scaffolds, and SH-SY5Y cells (**G**) alone and (**H**) S-SY5Y cells plus SW10 cells in soft and stiff scaffolds. White and black bars: soft and stiff scaffolds, respectively. Dashed lines indicate moving average per stiffness condition. X axis: time in weeks (w) and Y axis: % of VN stained area.

When SK-N-BE(2) cells were grown in 3D conditions, they reached high tVN expression levels (86.0 and 88.5% in soft and stiff hydrogels at the 2nd week) followed by a decrease in both soft and stiff conditions with just noticeable effect of stiffness on VN expression (Figure 4A). Although SH-SY5Y cells also increased their tVN expression when grown in 3D conditions (65.2 and 62.0% in soft and stiff hydrogels at the 2nd week), they mostly presented less tVN than SK-N-BE(2) cells. Interestingly, although SH-SY5Y stiff hydrogels followed a similar tVN expression dynamic as the SK-N-BE(2) ones over time, SH-SY5Y soft hydrogels slightly increased tVN expression from 2 to 6 weeks of cell culture (from 65.2 to 72.5%). In fact, at 6 weeks, there was a 30% difference in cell expression of tVN between soft and stiff scaffolds (Figure 4C).

SW10 cell co-culture reduced SK-N-BE(2) VN cell expression in all conditions when compared with SK-N-BE(2) cells grown alone. In both soft and stiff models VN expression was reduced from 2 to 6 weeks, particularly noticeable in the stiff ones. Remarkably, tVN expression increased at 8 weeks in cells grown in stiff hydrogels. In addition, SW10 cell co-culture contributed to the stiffness sensitivity of tVN malignant neuroblastic cell expression, which varied at each time point between soft and stiff hydrogels (Figure 4B). Adding SW10 cells also reduced SH-SY5Y cell VN expression, and as in SK-N-BE(2) cell plus SW10 cell hydrogels, soft and stiff models reduced in VN expression from 2 to 6 weeks with a final increase in tVN expression at 12 weeks in stiff hydrogels (Figure 4D). 

Regarding neuroblast cell population assessment at 6 weeks co-culture (performed by SYP staining (Figure 1B)), we found that the tVN area was reduced in both cell lines when compared with cell lines cultured alone in both soft and stiff conditions (52.6% expected vs. 20% observed in soft and 45.8% expected vs. 9% observed in stiff conditions for SK-N-BE(2) and 72.5% expected vs. 15% observed in soft and 42.7% expected vs. 15% observed in stiff conditions for SH-SY5Y) (Figure 1A). 

### 2.5. Co-Culture Condition with SW10 Modifies DOCK8 Expression Dynamics

Subjective quantification detected only very low levels of DOCK8 staining in both SK-N-BE(2) and SH-SY5Y 2D cell cultures and high expression in SW10 cells. 

**Figure 5:**
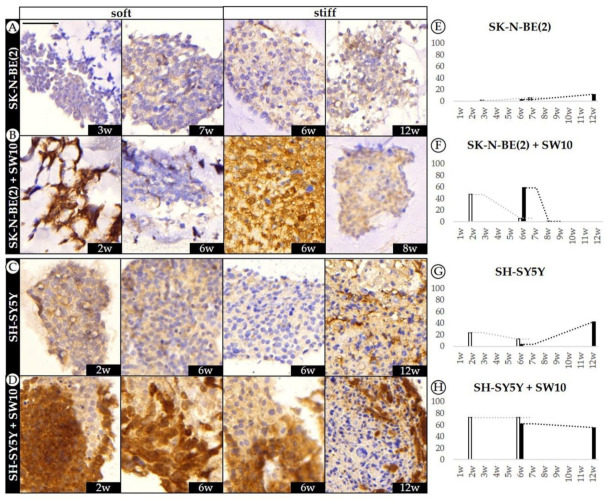
Dynamics of DOCK8 expression pattern in SK-N-BE(2) and SH-SY5Y over time. (**A**–**D**) Representative images of DOCK8 expression at the time points studied (w: weeks) in soft and stiff scaffoldings. Top images correspond to the SK-N-BE(2) cell line cultivated (**A**) alone and (**B**) with SW10; the images on the bottom represent the SH-SY5Y cell line cultivated (C) alone and (**D**) with SW10 cells. Scale bar 50 µm at top left of the first image. Same scale bar is valid for all images. (**E**–**H**) Bar chart quantification of DOCK8 staining (% of positive cells) for SK-N-BE(2) cells (**E**) alone and (**F**) SK-N-BE(2) cells plus SW10 cells in soft and stiff scaffolds, and SH-SY5Y cells (**G**) alone and (**H**) S-SY5Y cells plus SW10 cells in soft and stiff scaffolds. White and black bars: soft and stiff scaffolds, respectively. Dashed lines indicate moving average per stiffness condition. X axis: time in weeks (w) and Y axis: % of positive DOCK8 cells.

When we analyzed SK-N-BE(2) cells in 3D hydrogels, we also observed very low levels of DOCK8 expression, with an unexpected slight increase at 12 weeks in stiff hydrogels (Figure 5A). In addition, SH-SY5Y cells showed a remarkable increase in DOCK8 expression at 12 weeks in stiff hydrogels (42.2%), coinciding with the time point when there was almost no proliferation (0.2% of Ki67-positive cells). 

Co-culture of SK-N-BE(2) cells with SW10 cells led to greater expression of DOCK8 in the hydrogel cells, decreasing over time (from 47.3 to 6.1% in soft and 58.6 to 0.8% in stiff hydrogels) in which the soft hydrogels were affected earlier (Figure 5B). Finally, when comparing SH-SY5Y to SH-SY5Y plus SW10, we observed that over 50% of cells expressed DOCK8 at every given condition of the latter models. Furthermore, with stiffness and time, DOCK8 positive cells in SH-SY5Y plus SW10 cells model were prominent at the outer edge of many necrotic clusters (4D). 

### 2.6. Robust Expression of KANK1 in Models Including SH-SY5Y Cells Correlates Negatively with Proliferation

**Figure 6:**
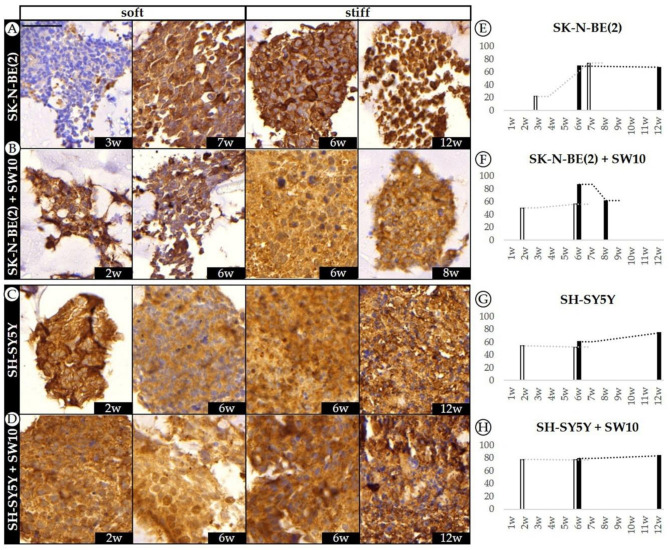
Dynamics of KANK1 expression pattern in SK-N-BE(2) and SH-SY5Y over time. (**A**–**D**) Representative images of KANK1 expression at the time points studied (w: weeks) in soft and stiff scaffoldings. The top images correspond to the SK-N-BE(2) cell line cultivated (**A**) alone and (**B**) with SW10; the images at the bottom represent the SH-SY5Y cell line cultivated (**C**) alone and (**D**) with SW10 cells. Scale bar 50 µm at top left of the first image. Same scale bar is valid for all images. (**E**–**H**) Bar chart quantification of KANK1 staining (% of positive cells) for SK-N-BE(2) cells (**E**) alone and (**F**) SK-N-BE(2) cells plus SW10 cells in soft and stiff scaffolds, and SH-SY5Y cells (**G**) alone and (**H**) S-SY5Y cells plus SW10 cells in soft and stiff scaffolds. White and black bars: soft and stiff scaffolds, respectively. Dashed lines indicate moving average per stiffness condition. X axis: time in weeks (w) and Y axis: % of KANK1 positive cells.

KANK1 was broadly expressed among NB cell lines SK-N-BE(2) and SH-SY5Y in 2D as well as in 3D hydrogels (Figure 6). SW10 cells also appeared positive in 2D cultures and 3D co-cultured hydrogels. 

In SK-N-BE(2) cells, KANK1 expression increased 52.2% from 3rd to 7th week (from 21.6 to 73.8%), unveiling the effect of culturing time on KANK1 immunostaining in soft hydrogels. However, KANK1 expression remained high when comparing stiff hydrogels from 6th to 12th week with a decrease of only 2% (from 69.0 to 67.0%). Moreover, no significant difference in KANK1 expression was observed at the 6th week due to stiffness variation (Figure 6A). In 3D cultured SH-SY5Y cells, the proportion of KANK1 highly positive cells was above 50% in all studied conditions, with a similar range to SK-N-BE(2) cells. We observed a slight increase of 8.6% in KANK1 expression comparing soft versus stiff hydrogels at 6 weeks (from 51.8 to 60.4% respectively). In addition, contrary to SK-N-BE(2) cell hydrogels, the proportion of KANK1 positive cells remained fairly constant over time in SH-SY5Y soft hydrogels (54.2 to 51.8% for 2 and 4 weeks, respectively) and displayed an increase of 13.8% of positive cells when grown in stiff hydrogels cultured up to 12 weeks (from 60.4 to 74.2%) (Figure 6C). 

On addition of SW10 cells to SK-N-BE(2) cell line cultures, the observed culturing time effect in soft SK-N-BE(2) hydrogels was lost and KANK1 expression increased only slightly from 50 to 56%. Moreover, comparing KANK1 expression at 6 weeks of growth, we observed increasing KANK1 positive cells from 56.0 to 86.9% with stiffness. Although KANK1 expression appeared higher in stiff hydrogels, there was a decrease in expression over time from 86.9 to 61.4% when compared at 6 and 8 weeks, respectively (Figure 6B). In SH-SY5Y cells co-cultured with SW10 cells, KANK1 expression was increased in comparison with SH-SY5Y hydrogels. In these models, 3D hydrogel stiffness only slightly modified the cell expression of KANK1 with variations not higher than 6% even when comparing extreme conditions (soft hydrogel at 2 weeks versus stiff hydrogel at 12 weeks) (Figure 6D). 

## 3. Discussion

Tumors are complex structures, made up of malignant cells coexisting within the stromal framework, including immune, lymphatic, and vascular endothelial cells [41,42]. The non-malignant cell population, together with the different molecules forming the stroma tissue such as GAGs, proteins, and glycoproteins, notably influence overall tumor cell behavior [42]. To study the effect of each of these components both individually and collectively, it is necessary to design artificial models that gradually increase in complexity. Three-dimensional bioprinting-related technologies already allow us to construct complex living structures with great precision, to the point of being able to generate tissue parts of functional organs such as the corneas or alveoli [43]. This technology is therefore ideal to improve current cancer modelling and for future development of artificial tumors with identical features to their in vivo counterparts. However, in order to faithfully recreate these tumors in 3D in vitro models, we must use biocompatible inks that enable not only malignant cell growth but also co-culture with non-malignant cells. The latter will contribute to synthesize and consolidate the tumor’s own matrix, which will ultimately replace the fabricated scaffold [44]. We generated different co-cultures with Schwann stromal cells as a first approach to mimicking NB complexity, which allowed us to evaluate the effect of the Schwann cells by comparison with the non-co-culture models. Moreover, these models are useful for conducting preclinical therapeutic trials, as they can be used to establish analyzable parameters through which to characterize the pathophysiological importance of tumor components and evaluate treatment responses [45]. As proof of concept, we selected Ki67, SYP, VN, DOCK8, and KANK1 expression for study, to give us an overview of tumor cell behavior from different biological perspectives, such as proliferation, aggressiveness, and migration-related protein synthesis, as well as consequences in terms of ECM remodeling and gene tumor suppression [27,28,33,38]. Finally, it is worth underlining the importance of having used analytical methodology to interpret the immunocytochemical data and to link it with previous genetic results; we chose digital image analysis, as it provides objective, quantifiable, and automatized information previously validated by the pathologist’s morphological assessment performed with digital imaging.

Ki67 evaluation revealed that SK-N-BE(2) cells proliferated more than SH-SY5Y cells on the models, indicating that the former exhibit a more aggressive phenotype. This is consistent with the presence of *MYCN* oncogene amplification in SK-N-BE(2) cells [46,47]. Moreover, the *ALK*-mutated SH-SY5Y cell line displayed low proliferation, but the cell population appeared similar to SK-N-BE(2), indicating good adaptation to the imposed growth conditions, since SH-SY5Y presented a lower karyorrhexis index than SK-N-BE(2), which could be related to an increased frequency of *ALK* mutations at a relapse of neuroblastoma. This finding highlights the importance of the ECM in tumor cell growth and behavior. Therefore, SH-SY5Y cells reach optimal growth in other models with different compositions [48,49] and including additional non-malignant cell populations in the models could help to generate the proper growth conditions for SH-SY5Y. SK-N-BE(2) and SH-SY5Y cells not only have diverse proliferation levels but also present dissimilar proliferation dynamics, since the substrate stiffness tends to increase proliferation in SK-N-BE(2) but not in SH-SY5. These data could be related to the differences observed in their genetic profile, which would indicate that stiffness has a strong effect on SK-N-BE(2). In fact, after 6 weeks of stiffness surroundings, new segmental chromosomal aberrations such as 9p (where DOCK8 and KANK1 genes are located) started to appear in SK-N-BE(2) but not in SH-SY5Y [22]. Further tests in other NB cell lines are warranted to evaluate whether the effect of stiffness on *MYCN*- and non-*MYCN*-amplified cells can be generalized. SW10 inclusion generally hindered proliferation in the models due to the fact that these cells are not as proliferative as NB tumor cells, and their presence thus reduced the overall proliferative index [50]. Nevertheless, differences found between the two co-cultures indicate that in soft hydrogels, SK-N-BE(2) cells can still grow and coexist with Schwann, but in stiff hydrogels, the Schwann cells overtake in proportion at 6 weeks of culture, with an increase in proliferative neuroblasts at 8 weeks of culture. Meanwhile, in SH-SY5Y cell co-cultures, there is a notable SW10-mediated proliferation decrease caused by the above-mentioned mechanisms. The data presented suggest that the stiffness of the 3D hydrogels could determine the degree to which Schwann cells affect malignant cell proliferation and karyorrhexis [49].

Cell growth is the result of excess proliferation over karyorrhexis. However, given the high SK-N-BE(2) proliferative indices, we would expect greater population growth than observed when compared to SH-SY5Y cultures. One plausible explanation could be different cell detachment from the scaffold occurring during cell cultures. In addition, the reduced proportion of cells present in highly proliferative models can be explained by processes such as maximal proliferation, reduced clone adaptation, or an asynchronous balance between proliferation and karyorrhexis and/or cellular metabolism, since metabolic reprograming must occur in order for cells to regulate cell growth, proliferation, and death [51]. Under this hypothesis, during the earliest cell culture time points, there would be a balance between proliferation of cells successfully adapting to the culture conditions and death of those unable to adapt. Subsequently, at longer cultivation times, cell metabolism would focus on other aggressive aspects such as migration, leaving proliferation aside. Nevertheless, this SK-N-BE(2) cell behavior would not accurately represent in vivo behavior without the presence of Schwann cells, highlighting the need to perform co-cultures in cancer models. Conversely, no correlation was found between proliferation and cell number in either SH-SY5Y model. Nonetheless, the time-related increase in cell numbers observed confirms the low cell proliferative index in these models and slow adaptation to imposed growth conditions, with less karyorrhexis and potentially lower cluster detachment events, which may account for the number of cells found in these models.

The high tVN expression found in 3D models demonstrates their usefulness compared to traditional models (VN expression in 2D cultures is scarce), as they better recreate the conditions found in vivo. The different tVN dynamics observed in 3D cultures seem to be determined by the specific needs of each cell line. Despite these slight variations, tVN expression is notable from the beginning of the cell culture in all the 3D models, exhibiting generally reduced (yet higher than in 2D culture) synthesis over time, which could be indicative of its key role not only in migration but also during the initial cell adaptation process. Interestingly, in the most clinically relevant models, those co-cultivated with SW10 cells, tVN expression levels are lower across all time points but increase at final culture times. This could be interpreted as an additional migration-related tVN role, which becomes apparent in those specific models, better recreating the metastatic pathways previously described in in vivo conditions [19].

Our results as regards DOCK8 expression dynamics support its cell migration role [31,32,33]. Data from SH-SY5Y cell and SH-SY5Y cell plus SW10 cell models correlate with a first stage of cell proliferation followed by a second stage where cells stop dividing in order to express migration-related mechanisms. Moreover, the elevated DOCK8 expression at longer culture time points in both SK-N-BE(2) and SH-SY5Y cells could also be indicative of cellular adaptation towards the migration process, which is further supported by the appearance of DOCK8 positive cells in the surroundings of necrotic clusters. On the other hand, taken together with Ki67 data, DOCK8 analysis in SW10 cell co-cultured models indicated that SK-N-BE(2) takes over in proportion in the conditions with low DOCK8 levels, as indicated by elevated proliferation and the proportion of SYP positive stained cells, which is also consistent with the fact that SK-N-BE(2) do not express high levels of DOCK8. When considering SH-SY5Y cell plus SW10 cell models, data confirm that SH-SY5Y cells have low proliferation capacity and that SW10 cells overtake in proportion. However, although we noted that SW10 cells expressed high amounts of DOCK8 in 2D cultures, we could not exclusively attribute the DOCK8 expression to SW10 cells, since some SH-SY5Y cells by themselves were also capable of expressing DOCK8. 

KANK1 expression was found to be highly expressed in every condition, in a stable and robust way. The few differences observed between models show that scaffold stiffness and SW10 inclusion play different roles depending on the cultured tumor cell line. Although the KANK1 data could support the notion of a cell migration stage following on from a cell adaptation and proliferation process, the few variations found in KANK1 expression, together with its multiple functions, necessitate functional studies to determine the specific role that this gene plays in NB tumors.

Taken together, the data herein show that physiologically relevant 3D tissue-engineered neuroblastoma cell models can be successfully developed and characterized using gelatin-based scaffolds, demonstrating their potential as tools to elucidate cell behavior and for new drug development. Performing co-cultures with SW10 has been shown to modify malignant cell action towards improved pathophysiological mimicking [50]. These models will also allow us to increase the complexity of the system by adding other cell types, such as mesenchymal stem cells or cells of the immune system. This creates a more physiologically relevant in vitro scenario [44], always provided that the presence and behavior of each cell group included in the model can be identified and monitored. Further, the influence of cellular genetics during model development can be assessed in these platforms to predict the behavior of similar patient-derived cells. In the future, when optimal scaffolds can be generated for patient cell growth, these specifically adapted scaffold conditions will be required to carry out the corresponding preclinical therapeutic studies.

## 4. Materials and Methods

### 4.1. Cell Culture

SK-N-BE(2), SH-SY5Y human NB cell lines were chosen from a variety of available cell lines, since *MYCN*-amplified and *ALK*-mutated tumors represent 64% of high risk neuroblastoma (50 and 14%, respectively). SK-N-BE(2), SH-SY5Y, and SW10 mouse Schwann cell lines were acquired from American Type Culture Collection (ATCC, Manassas, VA, USA). NB cells were expanded in supplemented IMDM medium (Gibco, Life Technologies, Waltham, MA, USA) and SW10 in supplemented DMEM (Gibco, Life Technologies, Waltham, MA, USA) at 37 °C and 5% CO2 atmosphere. Two dimensional cell cultures were grown in 8-well Cell Culture Slides (SPL Life Sciences, Waltham, MA, USA), until they reached 60% confluence before immunocytochemistry (ICC) analysis. Bioinks for 3D culture were formed by mixing SK-N-BE(2) or SH-SY5Y cells with the prepolymer solution at 37 °C to a final solution of 2 × 10^6^ mL^−1^, as previously reported [21], including in some cases, an additional 2 × 10^5^ mL^−1^ of SW10 cells. Hydrogels were cultured from 2 to 12 weeks in supplemented IMDM medium replaced every 2 or 3 days before immunohistochemistry (IHC) analysis.

### 4.2. Synthesis of Hydrogels

Composite hydrogels were synthetized using gelatin and alginate, as previously described [21,52]. Prepolymer solutions were prepared to obtain final concentrations of 5% *w*/*v* methacrylated gelatin (GelMA) and 0–0.5% (soft) or 1.5–2% (stiff) methacrylated alginate (AlgMA), according to the desired initial stiffness level. The bioink with the cells was loaded in a bioprinting syringe and gelified at −20 °C for 3 min before printing. All hydrogels were fabricated using a 3D bioprinter (3DDiscovery BioSafety, RegenHU, Villaz-St-Pierre, Switzerland; 365 nm, 3 W cm^−2^) and polymerized with UV light, as previously reported [21,52]. 

### 4.3. Immunocytochemistry and Immunohistochemistry

ICC stains were performed with anti-Ki67 (prediluted, Agilent Technologies, Santa Clara, CA, USA), anti-VN (1/100, Abcam, Cambridge, UK), anti-DOCK8 (1/700, Invitrogen, Life Technologies, Waltham, MA, USA), anti-KANK1 (1/1000, Invitrogen, Life Technologies, USA), and anti-SYP (prediluted, Dako, Glostrup, Denmark) antibodies. Anti-Ki67 labeling (nuclear staining) (Autostainer Link 48; Dako, Glostrup, Denmark), anti-VN labeling (cytoplasm and extracellular staining) (BenchMark XT, Ventana Medical Systems Inc., Tucson, AZ, USA), and anti-SYP labeling (cytoplasm staining) (Autostainer Link 48; Dako, Glostrup, Denmark) were automatically performed, while anti-DOCK8 (membrane, cytoplasm, and nuclear staining in normal cells) and anti-KANK1 (membrane, cytoplasm, and nuclear staining in normal cells) were done manually. Four percent formaldehyde and paraffin-embedded hydrogels were cut into 3 μm sections. IHC stains were performed as indicated above, including an antigen retrieval step using the corresponding buffer for each antibody (PT Link instrument, Agilent, Santa Clara, CA, USA). All markers were evaluated by optical microscopy using the following criteria: negative (−, <1% positive cells); low positive (+, 1–20% positive cells); intermediate positive (++, 20–50% positive cells); high positive (+++, >50% positive cells); and by digital image analysis. Hematoxylin–eosin stained samples were used to evaluate karyorrhexis index and cell detachment from 3D hydrogels (after trypsinization of the wells used for 3D cultures) by optical microscopy evaluation.

### 4.4. Image Analysis

IHC stained slides were digitized with the whole-slide Pannoramic MIDI scanner (3DHISTECH Ltd., Budapest, Hungary) at 20× magnification. Digital image analysis was performed on whole sample areas, excluding only detected artefacts and folded and/or broken regions, which were considered uninformative. Ki67, SYP, KANK1, and DOCK8 were analyzed automatically using Pannoramic Viewer (PV) software (3DHISTECH), and their number of positive cells were determined by applying the NuclearQuant module. This module could be applied to non-nuclear stained samples by adjusting nucleus size settings to detect entire cells when required (nuclear radius 3–15 µm) and adjusting color deconvolution settings. HistoQuant module of PV was applied in VN-stained sections to obtain the areas of each VN intensity expression. All data obtained from PV modules were validated with pathologist’s morphological assessment of the digital image. Digitally obtained data and subsequent pathologist evaluation differed by only 5–10%.

### 4.5. Data Treatment

For Ki67, SYP, DOCK8, and KANK1 expression-related analysis, digital positive cell percentages were compared for each condition under study. We calculated the percentage of positive cells in each sample, considering as positive cells only those with digitally detected medium and high intensity expression. By considering low intensity expression as negative cells, we avoided including artifacts and unspecific staining areas in the analysis. 

As previously described in our group [20], tVN intensity is higher than interterritorial VN. The tVN quantification was performed by calculating the percentage of strong staining intensity, territorial location, and H-score ≥ 180 from the total VN stain according to PV detection. Population cell growth was assessed correlating cell proliferative index (Ki67 positivity), cell number (number of cells, standardized by hydrogel area in mm^2^ for comparison between conditions), and culturing time. 

In order to characterize the SW10 vs. the neuroblast population, we used SYP as a differential marker between SW10 and neuroblastic cells since both SK-N-BE(2) and SH-SY5Y cell lines were 100% positive and SW10 negative. Since Ki67 and tVN are expressed only by the NB cells, we assumed that all the quantified staining belonged to NB cells. Therefore, we used the percentage of SYP to normalize the neuroblast population in co-cultured models and assessed the specific effect of SW10 in the neuroblastic cells (Figure 1A). 

## 5. Conclusions

The tools presented in this study provide the data for successful development and characterization of pathophysiologically relevant 3D tissue-engineered neuroblastoma cell models. Through 3D bioprinted gelatin-based scaffolds, we were able to determine and study the effect of stiffness on SK-N-BE(2) and SH-SY5Y neuroblastoma cell lines. We generated the models using bioinks in order to study the more complex spheroid cells. Adding stromal SW10 cells in the bioink allowed co-culture with malignant neuroblastic cells, creating a biocompatible niche where cells could grow over long periods of time and recapitulate tumor-specific organization. We present robust and detailed phenotypic characterization of 3D models, based on objective immunostaining quantification using digital image analysis pipelines. As proof of concept, we selected Ki67, SYP, VN, DOCK8, and KANK1 expression, which are pivotal for study, as they provide an overview of tumor cell behavior from different biological perspectives such as proliferation, aggressiveness, and migration-related protein synthesis and consequences in terms of ECM remodeling and gene tumor suppression. 

## Abbreviations

ECM	extracellular matrix
IHC	immunohistochemistry
PV	Pannoramic Viewer
SW10	mouse Schwann cell line
SYP	synaptophysin
NB	neuroblastoma
VN	vitronectin
2D	2 dimensional
3D	3 dimensional
